# Intercomparison of diffusion coefficient derived from the through-diffusion experiment using different numerical methods

**DOI:** 10.1007/s10967-014-2974-8

**Published:** 2014-02-02

**Authors:** Chih-Lung Chen, Tsing-Hai Wang, Ching-Hor Lee, Shi-Ping Teng

**Affiliations:** 1 Department of Engineering and System Science, National Tsing Hua University, Hsinchu, Taiwan; 2 Division of Chemical Engineering, Institute of Nuclear Energy Research, Taoyuan, Taiwan; 3 Department of Medical Imaging and Radiological Sciences, I-Shou University, Kaohsiung, Taiwan

**Keywords:** Decay effect, Through-diffusion, Diffusion coefficient, Radionuclide

## Abstract

Diffusion is a dominant mechanism regulating the transport of released nuclides. The through-diffusion method is typically applied to determine the diffusion coefficients (D). Depending on the design of the experiment, the concentrations in the source term [i.e., inlet reservoir (IR)] or the end term [i.e., outlet reservoir (OR)] can be fixed or vary. The combinations involve four distinct models (i.e., the CC–CC model, CC–VC model, VC–CC model, and the VC–VC model). Studies discussing the VC–CC model are scant. An analytical method considering the decay effect is required to accurately interpret the radioactive nuclide diffusion experiment results. Therefore, we developed a CC–CC model and a CC–VC model with a decay effect and the simplified formulas of these two models to determine the diffusion coefficient (i.e., the CC–CC method and CC–VC method). We also proposed two simplified methods using the VC–VC model to determine the diffusion coefficient straightforwardly based upon the concentration variation in IR and OR. More importantly, the best advantage of proposed method over others is that one can derive three diffusion coefficients based on one run of experiment. In addition, applying our CC–VC method to those data reported from Radiochemica Acta 96:111–117, 2008; and J Contam Hydrol 35:55–65, 1998, derived comparable diffusion coefficient lying in the identical order of magnitude. Furthermore, we proposed a formula to determine the conceptual critical time (*T*c), which is particularly beneficial for the selection of using CC–VC or VC–VC method. Based on our proposed method, it becomes possible to calculate diffusion coefficient from a through-diffusion experiment in a shorter period of time.

## Introduction

Diffusion is a dominant mechanism regulating the transport of released nuclides from the near field of the final disposal repository site. Diffusion constants are typically obtained using diffusion experiments. Among the numerous techniques currently available, the through-diffusion method is popularly applied for determining the diffusion coefficients [1, 2]. The through-diffusion method is applied when the geological medium (i.e., specimen) is surrounded by two reservoirs, where one reservoir contains a concentration of nuclide (i.e., the source term) and the other reservoir is nuclide-free (i.e., the end term). The source term is known as an injective reservoir or inlet reservoir (IR), and the end term is known as a diffusive reservoir (DR) or outlet reservoir (OR).

Depending on the design of the experiment, the concentration in the source term can be fixed (i.e., a constant concentration source) or vary (i.e., a variable concentration source). Similarly, the concentration in the end term can be fixed or variable. The combinations involve a constant inlet concentration–constant outlet concentration model (CC–CC model) [3, 4], a constant inlet concentration–variable outlet concentration model (CC–VC model) [5, 6], a variable inlet concentration–constant outlet concentration model (VC–CC model) [3], and a variable inlet concentration–variable outlet concentration model (VC–VC model) [7–9]. The estimated methods of the diffusion coefficients corresponding to the models are termed the CC–CC method, CC–VC method, VC–CC method, and VC–VC method, respectively. Among these models, the VC–CC model is rarely designed and performed. Certain studies have presented overviews and discussed the differences of those models, and the diffusion coefficient estimated methods were also provided [2, 10, 11]. However, these studies have not considered the decay effect in the radioactive nuclide diffusion experiment. The diffusion experiment is typically time-consuming, and the decay effect should be considered. Each experiment is necessary for a reasonable parameter estimation method [10]. In this study, we developed a CC–CC model and a CC–VC model with decay effect, as well as a parameter estimation method (CC–CC method and CC–VC method) for helping calculate diffusion coefficient by experimental researchers.

Experimental researchers specialize in experimental operations, and may overlook the fact that parameter estimation should be coordinated with the design of the experiment. For example, the design of the experiment may require a CC–VC model, but the CC–CC method is applied for parameter estimation. A significant difference exists in the intrinsic diffusion coefficient derived using varied through-diffusion solution methods. For this issue, we discussed the differences from various methods for distinct models by using numerical experiments [12].

In the VC–VC model, after laborious and time-consuming one only gains a diffusion coefficient value from measuring the nuclide concentration difference between IR and OR. This is currently a resources waste. The literatures [7–9] did not apply those useful data. Thus, we develop the diffusion coefficient estimated methods from the concentration distributions of the IR and OR in the VC–VC model. This benefits for cross comparison of the estimated diffusion coefficient and enhances experiment effectiveness.

## Methodology

A 1D diffusion equation derived from mass balance is adopted to describe the solute diffusion transport in the porous medium by using the following equation

1 $$ D_{p} \frac{{\partial^{2} C}}{{\partial x^{2} }} - \frac{{\rho_{b} }}{n}\frac{\partial S}{\partial t} = \frac{\partial C}{\partial t} $$

where *C* is the solute concentration in the pore water [M/L^3^]; *D*_p_ is theIntrinsic diffusion coefficient in the pore water [L^2^/T]; *S* is the mass of solute absorbed per unit bulk dry mass of the porous medium [–]; *n* is the porosity of the porous medium [–]; *ρ*_b_ is the bulk dry density of the porous medium [M/L^3^]; *x* is the length coordinate [L]; *t* is the time [T]

The first term on the left-hand side of (1) describes diffusion in the mobile pore water. The second term describes the solute absorbed by the medium. The term on the right-hand side of (1) describes the accumulation of the solute.

Assuming that sorption follows a linear relationship of $S = K_{\rm d} C$, where* K*_d_ is the distribution coefficient [L^3^/M], (1) can be reduced to

2 $$ D_{p} \frac{{\partial^{2} C}}{{\partial x^{2} }} = \frac{\partial C}{\partial t}(1 + \frac{{\rho_{b} }}{n}K_{d} ) $$

or

3 $$ D\frac{{\partial^{2} C}}{{\partial x^{2} }} = \frac{\partial C}{\partial t} $$

where $D$ is the apparent diffusion coefficient [L^2^/T] [$D$ can be expressed as $D = \frac{{D_{p} }}{R}$, where $R = 1 + \frac{{\rho_{b} }}{n}K_{d}$ is the retardation factor (–)].

Considering that the dissolved solute is a radioactive nuclide, (3) adds a decay term and becomes

4 $$ D\frac{{\partial^{2} C}}{{\partial x^{2} }} - \lambda C = \frac{\partial C}{\partial t} $$

where $\lambda$ is the decay constant [1/*T*] (which can be expressed as $\lambda = {{\ln \left( 2 \right)} \mathord{\left/ {\vphantom {{\ln \left( 2 \right)} {H_{\text{f}} }}} \right. \kern-0pt} {H_{\text{f}} }}$; $H_{\text{f}}$ is the half-life [*T*]).

Equation (4) is the governing equation of the 1D diffusion model with the decay effect.

The initial equation is

5 $$ C\left( {x,t = 0} \right) = 0 $$

Various diffusion experiment types have different assumptions of boundary conditions and estimated methods for diffusion coefficients.

### CC–CC model

#### Experimental concept

The boundaries of the CC–CC model with the consideration of decay effect are

6 $$ \, \left\{ {\begin{array}{*{20}c} {C\left( {x = 0,t} \right) = C_{o} {\text{e}}^{ - \lambda t} } \hfill \\ {C\left( {x = L,t} \right) = 0} \hfill \\ \end{array} } \right. $$

where *C*_o_ is the initial concentration in the IR [M/L^3^], and *L* is the thickness of the specimen [*L*].

**Fig. 1:**
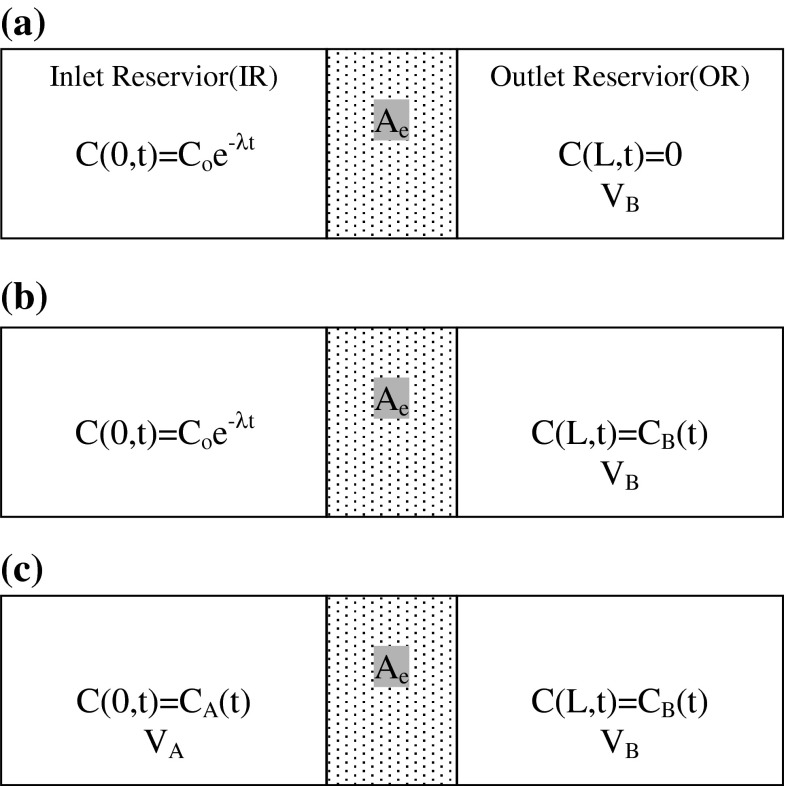
Schematic diagram of three various through-diffusion models. **a** CC–CC Model, **b** CC–VC Model,** c** VC–VC Model

The experimental concept of the CC–CC model is demonstrated in Fig. 1a. The solute concentration in the IR (left-hand side) is assumed constant while the solute concentration in the OR (right-hand side) should always be zero all the time. However, when conducting a diffusion experiment, the solute concentration in the IR would actually decrease because the radioactive nuclides will decay and diffuse into the media. The solute concentration in the right-hand side (OR) would change as well because certain amount of solute have diffused through the media. In order to keep the experimental conditions meet the boundary condition of CC–CC model, one must periodically add additional solute into IR and periodically replace the solution in the OR. From this point of view, a practical alternative is to greatly increase the volume of both IR and OR so that the varying solute concentration may be reasonably assumed negligible.

#### Analytical solution

The solute migration equation of the CC–CC model in the OR is [3]

7 $$ C = C_{o} \left( {1 - \frac{x}{L} - \sum\limits_{n = 1}^{\infty } {\frac{2}{n\pi }\sin \frac{n\pi x}{L}{\text{e}}^{{ - \frac{{Dn^{2} \pi^{2} }}{{L^{2} }}t}} } } \right) $$

where *n* = 1, 2, …, ∞.

With the decay effect, the equation can be expressed as a multiple of ${\text{e}}^{ - \lambda t}$; that is,

8 $$ C = C_{o} \left( {1 - \frac{x}{L} - \sum\limits_{n = 1}^{\infty } {\frac{2}{n\pi }\sin \frac{n\pi x}{L}{\text{e}}^{{ - \frac{{Dn^{2} \pi^{2} }}{{L^{2} }}t}} } } \right){\text{e}}^{ - \lambda t} $$

Equation (8) satisfies the governing Eq. (4), initial condition (5), and boundary conditions (6). This proves that (8) is the analytical solution of the CC–CC model with a decay effect.

#### CC–CC method for estimating D

The procedures for estimating the apparent diffusion coefficient of the CC–CC model with a decay effect are as follows. The flux at *x* = *L* can be expressed by

9 $$ J(L,t) = \left. { - D\frac{\partial C}{\partial x}} \right|_{x = L} = C_{o} \frac{D}{L}\left[ {1 + 2\sum\limits_{n = 1}^{\infty } {( - 1)^{n} {\text{e}}^{{ - \frac{{Dn^{2} \pi^{2} }}{{L^{2} }}t}} } } \right]{\text{e}}^{ - \lambda t} $$

The total quantity, Q(t), diffused through the media with an effective cross-area (*A*_e_) of up to time t can be calculated as

10 $$ \begin{aligned} Q(t) & = \int_{0}^{t} {J\left( {L,t} \right)A_{\text{e}} dt} = \frac{{DA_{\text{e}} C_{o} }}{L}\left\{ { - \frac{1}{\lambda }{\text{e}}^{ - \lambda t} + \frac{L}{{2\sqrt {\lambda D} }}\left[ {\coth \left( {\frac{L}{2}\sqrt {\frac{\lambda }{D}} } \right) - \tanh \left( {\frac{L}{2}\sqrt {\frac{\lambda }{D}} } \right)} \right]} \right. \\ & - \left. {2\sum\limits_{n = 1}^{\infty } {\frac{{( - 1)^{n} }}{{\frac{{Dn^{2} \pi^{2} }}{{L^{2} }} + \lambda }}{\text{e}}^{{ - \left( {\frac{{Dn^{2} \pi^{2} }}{{L^{2} }} + \lambda } \right)t}} } } \right\} \\ \end{aligned} $$

From the point of view of diffusion experiments, the concentration of solute that has diffused through the media in the OR should be determined during each solution replacement. While the solute concentration ratio into the OR can be thus experimentally determined, the numerical concentration ratio in the OR can be expressed as

11 $$ \begin{aligned} \frac{C(t)}{{C_{\text{o}} }} = \frac{Q(t)}{{V_{\text{B}} }} & = \frac{{DA_{\text{e}} }}{{V_{\text{B}} L}}\left\{ { - \frac{1}{\lambda }{\text{e}}^{ - \lambda t} + \frac{L}{{2\sqrt {\lambda D} }}\left[ {\coth \left( {\frac{L}{2}\sqrt {\frac{\lambda }{D}} } \right) - \tanh \left( {\frac{L}{2}\sqrt {\frac{\lambda }{D}} } \right)} \right]} \right. \\ & - \left. {2\sum\limits_{n = 1}^{\infty } {\frac{{( - 1)^{n} }}{{\frac{{Dn^{2} \pi^{2} }}{{L^{2} }} + \lambda }}{\text{e}}^{{ - \left( {\frac{{Dn^{2} \pi^{2} }}{{L^{2} }} + \lambda } \right)t}} } } \right\} \\ \end{aligned} $$

where *V*_B_ is the OR volume [L^3^].

As t increases, the third term in (11) decreases rapidly to yield the asymptotic solution $C'(t)$

12 $$ \frac{C'(t)}{{C_{\text{o}} }} = \frac{{DA_{\text{e}} }}{{V_{\text{B}} L}}\left\{ { - \frac{1}{\lambda }{\text{e}}^{ - \lambda t} + \frac{L}{{2\sqrt {\lambda D} }}\left[ {\coth \left( {\frac{L}{2}\sqrt {\frac{\lambda }{D}} } \right) - \tanh \left( {\frac{L}{2}\sqrt {\frac{\lambda }{D}} } \right)} \right]} \right\} $$

Equation (12) is a straight line of slope $s = - \frac{{DA_{\text{e}} }}{{\lambda V_{\text{B}} L}}$ with ${\text{e}}^{ - \lambda t}$. Therefore, the values of the apparent diffusion coefficient can be determined as

13 $$ D = - \frac{{s\lambda V_{\text{B}} L}}{{A_{\text{e}} }} $$

### CC–VC model

#### Experimental concept

The CC–VC model is a popular diffusion experiment model. The initial conditions are (5), and the equations are as follows:

14 $$ t = 0, \, \left\{ {\begin{array}{*{20}c} {C\left( {x = 0,t} \right) = C_{o} } \hfill \\ {C\left( {x = L,t} \right) = C_{\text{B}} \left( t \right) = 0} \hfill \\ \end{array} } \right. $$

where C_B_ represents the concentration in the OR [M/L^3^].

The boundary conditions with a decay effect are expressed as

15 $$ x = 0, \, C\left( {x = 0,t} \right) = C_{o} {\text{e}}^{ - \lambda t} $$

16 $$ x = L, \, - \lambda V_{\text{B}} C_{\text{B}} - A_{e} D\left. {\frac{\partial C}{\partial x}} \right|_{x = L} = V_{\text{B}} \frac{{{\text{d}}C_{\text{B}} }}{{{\text{d}}t}} $$

Figure 1b shows the experimental concept of the CC–VC model. The constant concentration in the IR decreases with the decay effect. In a practical experiment, one increases the volume or the initial concentration in the IR similar to the CC–CC model for increasing the solute in the IR. In the OR, the concentration increases with time, which is different from the CC–CC model (in which the OR is maintained at a zero concentration). The concentration in the OR is measured periodically during the experiment.

#### Solution

1. Semi-analytical solution

The Laplace transform method was used to develop the concentration formula with a decay effect for the specimen and the OR. The concentrations in the Laplace domain are

17 $$ \overline{C} \left( {x,p} \right) = \frac{{C_{\text{o}} }}{p + \lambda }\left[ {\cosh (mx) - \frac{M\sinh (mL) + \cosh (mL)}{M\cosh (mL) + \sinh (mL)}\sinh (mx)} \right] $$

18 $$ \overline{{C_{\text{B}} }} \left( p \right) = \frac{{C_{\text{o}} }}{p + \lambda }\frac{M}{M\cosh (mL) + \sinh (mL)} $$

where $$ M = \frac{{mA_{\text{e}} D}}{{V_{\text{B}} (p + \lambda )}} $$ and $$ m = \sqrt {\frac{p + \lambda }{D}} $$.

Equations (17) and (18) respectively satisfy the governing Eq. (4), initial condition (5), and boundary conditions (15) and (16) in the Laplace domain. These prove that (17) is a semi-analytical solution of the CC–VC model, and that (18) is the concentration formula in the OR.

(2) Analytical solution

Similar to the discussion by Chen et al. [9], the multi-compartment (MC) model was used to develop the analytical solution of the CC–VC model with a decay effect. The derivation steps are presented below.

Starting with the consideration of Fick laws, ($J = - D\frac{\partial C}{\partial x}$), together with the assumption that the specimen was adjacent to one compartment, the mass balance of the nuclide between each compartment is derived and rearranged as follows:

19 $$ \left\{ {\begin{array}{*{20}c} {V_{1} \frac{{{\text{d}}C}}{{{\text{d}}t}} = 2\alpha C_{\text{B}} - \left( {4\alpha + \lambda V_{1} } \right)C + 2\alpha C_{\text{o}} e^{ - \lambda t} } \hfill \\ {V_{\text{B}} \frac{{{\text{d}}C_{\text{B}} }}{{{\text{d}}t}} = 2\alpha C - \left( {2\alpha + \lambda V_{\text{B}} } \right)C_{\text{B}} } \hfill \\ \end{array} } \right. $$

where $V_{1} = A_{\text{e}} L$ and $\alpha = \frac{{A_{\text{e}} D}}{L}.$ Using the Laplace transform and substituting the following initial conditions yields

$$ t \, = \, 0,\,\,C_{\text{B}} = \, C \, = \, 0 $$

These equations can be solved in algebraic form as

20 $$ \left\{ {\begin{array}{*{20}c} {\overline{C}_{\text{B}} = \frac{2\alpha }{{2\alpha + (p + \lambda )V_{\text{B}} }}\overline{C} } \hfill \\ {\overline{C} = \frac{{\frac{2\alpha }{p + \lambda }}}{{4\alpha + (p + \lambda )V_{1} - \frac{{\left( {2\alpha } \right)^{2} }}{{2\alpha + (p + \lambda )V_{\text{B}} }}}}C_{\text{o}} } \hfill \\ \end{array} } \right. $$

The Laplace inverse transformations of these equations were implemented using the symbolic-numerical software package Mathematica 8.0 [13]. The solutions obtained under the real-time domain are as follows:

 In the specimen:

21 $$ C = C_{\text{o}} \left[ {{\text{e}}^{ - \lambda t} + \frac{{\alpha V_{1} }}{2\beta }\left( {\gamma_{1} - \gamma_{2} } \right) - \frac{1}{2}\left( {\gamma_{1} + \gamma_{2} } \right)} \right] $$

In the OR:

22 $$ C_{\text{B}} = C_{\text{o}} \left[ {{\text{e}}^{ - \lambda t} + \frac{\alpha }{2\beta }\left( {V_{1} + 2V_{\text{B}} } \right)\left( {\gamma_{1} - \gamma_{2} } \right) - \frac{1}{2}\left( {\gamma_{1} + \gamma_{2} } \right)} \right] $$

where

$$ \gamma_{1} = {\text{e}}^{{ - \left( {\lambda + \frac{2\alpha }{{V_{1} }} + \frac{\alpha }{{V_{\text{B}} }} + \frac{\beta }{{V_{1} V_{\text{B}} }}} \right)t}} ,\,\gamma_{2} = {\text{e}}^{{ - \left( {\lambda + \frac{2\alpha }{{V_{1} }} + \frac{\alpha }{{V_{\text{B}} }} - \frac{\beta }{{V_{1} V_{\text{B}} }}} \right)t}} ,\,{\text{and}}\,\beta = \sqrt {\alpha^{2} \left( {V_{1}^{2} + 4V_{\text{B}}^{2} } \right)} . $$

#### CC–CV method for estimating D

Without the decay effect of the CC–VC model, an proposed asymptotic solution for estimating D by using the following expression [14]:

23 $$ \frac{{C_{\text{B}} (t)}}{{C_{\text{o}} }} = 1 - {\text{e}}^{{ - \frac{{A_{\text{e}} D}}{{V_{\text{B}} L}}t}} $$

This formula works only when the decay effect is negligible. We incorporated the decay effect into this study as shown in the analytical solutions (22). The derivation procedures are as follows:

First, we assume that *V*_B_ ≫ *V*_1_ and *t* ≫ 0. Then,

$$ \begin{gathered} \beta = \sqrt {\alpha^{2} \left( {V_{1}^{2} + 4V_{\text{B}}^{2} } \right)} \approx 2\alpha V_{\text{B}} , \hfill \\ \gamma_{1} = {\text{e}}^{{ - \left( {\lambda + \frac{2\alpha }{{V_{1} }} + \frac{\alpha }{{V_{\text{B}} }} + \frac{\beta }{{V_{1} V_{\text{B}} }}} \right)t}} \approx 0, \hfill \\ \gamma_{2} = {\text{e}}^{{ - \left( {\lambda + \frac{2\alpha }{{V_{1} }} + \frac{\alpha }{{V_{\text{B}} }} - \frac{\beta }{{V_{1} V_{\text{B}} }}} \right)t}} \approx {\text{e}}^{{ - \left( {\lambda + \frac{\alpha }{{V_{\text{B}} }}} \right)t}} . \hfill \\ \end{gathered} $$

Therefore,

24 $$ \frac{{C_{\text{B}} (t)}}{{C_{o} }} = \left( {1 - {\text{e}}^{{ - \frac{{A_{\text{e}} D}}{{V_{\text{B}} L}}t}} } \right){\text{e}}^{ - \lambda t} $$

After arrangement, this can be expressed as

25 $$ \ln \left[ {\frac{{C_{o} - C_{\text{B}} (t) \cdot {\text{e}}^{\lambda t} }}{{C_{\text{o}} }}} \right] = - \frac{{A_{\text{e}} D}}{{V_{\text{B}} L}}t $$

Equation (25) shows that $\ln \left[ {\frac{{C_{o} - C_{\text{B}} (t) \cdot {\text{e}}^{\lambda t} }}{{C_{\text{o}} }}} \right]$ varies with t with a constant slope (s). Therefore, D can be determined as

26 $$ D = - \frac{{sV_{\text{B}} L}}{{A_{\text{e}} }} $$

### VC–VC model

#### Experimental concept

The initial condition of the VC–VC model includes (5) and the following equations:

27 $$ t = 0, \, \left\{ {\begin{array}{*{20}c} {C\left( {x = 0,t} \right) = C_{\text{A}} \left( t \right) = C_{o} } \hfill \\ {C\left( {x = L,t} \right) = C_{\text{B}} \left( t \right) = 0} \hfill \\ \end{array} } \right. $$

where C_A_ represents the concentration in the IR [M/L^3^].

The boundary conditions with the decay effect are expressed as

28 $$ x = 0, \, \left\{ {\begin{array}{*{20}c} {C\left( {x = 0,t} \right) = C_{\text{A}} \left( t \right) = C_{\text{o}} \text{e}^{ - \lambda t} } \hfill \\ { - \lambda V_{\text{A}} C_{\text{A}} + A_{\text{e}} D\left. {\frac{\partial C}{\partial x}} \right|_{x = 0} = V_{\text{A}} \frac{{{\text{d}}C_{\text{A}} }}{{{\text{d}}t}}} \hfill \\ \end{array} } \right. $$

29 $$ x = L, \, \left\{ {\begin{array}{*{20}c} {C\left( {x = L,t} \right) = C_{\text{B}} \left( t \right)} \hfill \\ { - \lambda V_{\text{B}} C_{\text{B}} - A_{\text{e}} D\left. {\frac{\partial C}{\partial x}} \right|_{x = L} = V_{\text{B}} \frac{{{\text{d}}C_{\text{B}} }}{{{\text{d}}t}}} \hfill \\ \end{array} } \right. $$

where *V*_A_ is the volume of the IR [L^3^].

The experimental concept is similar to that showed in Fig. 1c. The known concentration is injected into the IR from the beginning of the experiment. The concentration in the IR declines with the decay effect and diffuses through the specimen into the OR. The concentration in the OR varies with time. During the experiment, maintaining a constant concentration or increasing the solute in the IR is unnecessary. In addition, one only periodically measures concentrations in the IR and OR during the experiment. Measuring the IR concentration is the major difference between this model and the CC–VC model. Although the measuring process of the concentration of the IR may spend resources, one can successfully estimate and confirm the diffusion coefficient, which is discussed in the following section.

#### Solution

1. Semi-analytical solution

The VC–VC model with a decay effect can refer to the Ref. [9]. The semi-analytical solutions using the advection–dispersion (AD) model in the Laplace domain are as follows:

30 $$ \overline{C}_{\text{A}} \left( p \right) = \frac{{M_{2} V_{\text{A}} C_{\text{o}} }}{{(p + \lambda )M_{2} V_{\text{A}} - (p + \lambda )M_{1} V_{\text{B}} }} $$

31 $$ \overline{C}_{\text{B}} \left( p \right) = \frac{{V_{\text{A}} C_{o} }}{{(p + \lambda )M_{2} V_{\text{A}} - (p + \lambda )M_{1} V_{\text{B}} }}\left[ {M_{2} \cosh (m_{1} L) + \sinh (m_{1} L)} \right] $$

where $$ M_{2} = - \frac{{M_{1} \cosh (m_{1} L) + \sinh (m_{1} L)}}{{M_{1} \sinh (m_{1} L) + \cosh (m_{1} L)}} $$, $$ M_{1} = \frac{{m_{1} A_{\text{e}} D}}{{V_{\text{B}} (p + \lambda )}} $$, and $$ m_{1} = \sqrt {\frac{p + \lambda }{D}} $$.

(2) Analytical solution

The analytical solutions using the multi-compartment (MC) model in the IR and OR are as follows:

In the IR:

32 $$ \begin{aligned} C_{\text{A}} & = \frac{{V_{\text{A}} C_{\text{o}} }}{V}\left\{ {\text{e}^{ - \lambda t} + \frac{{\left( {\gamma_{3} - \gamma_{4} } \right)\left( { - V_{1}^{2} V_{\text{A}} + 2V_{1}^{2} V_{\text{B}} - V_{1} V_{\text{A}} V_{\text{B}} + V_{1} V_{\text{B}}^{2} - 2V_{\text{A}} V_{\text{B}}^{2} } \right)}}{{2\beta_{1} V_{\text{A}} }}} \right. \\ & + \left. {\frac{{\beta_{1} \left( {\gamma_{3} + \gamma_{4} } \right)\left( {V_{1} + V_{\text{B}} } \right)}}{{2\beta_{1} V_{\text{A}} }}} \right\} \\ \end{aligned} $$

In the OR:

33 $$ C_{\text{B}} = \frac{{V_{\text{A}} C_{\text{o}} }}{V}\left\{ {{\text{e}}^{ - \lambda t} + \frac{{\left( {\gamma_{3} - \gamma_{4} } \right)\left( {V_{1} V_{\text{A}} + V_{1} V_{\text{B}} + 2V_{\text{A}} V_{\text{B}} } \right) - \beta_{1} \left( {\gamma_{3} + \gamma_{4} } \right)}}{{2\beta_{1} }}} \right\} $$

where

$$ \begin{gathered} V = V_{\text{A}} + V_{1} + V_{\text{B}} ,\,\,\beta_{1} = \sqrt {V_{1}^{2} V_{\text{A}}^{2} - 2V_{1}^{2} V_{\text{A}} V_{\text{B}} + V_{1}^{2} V_{\text{B}}^{2} + 4V_{\text{A}}^{2} V_{\text{B}}^{2} } , \hfill \\ \gamma_{3} = {\text{e}}^{{ - [\lambda + \alpha (\frac{1}{{V_{\text{A}} }} + \frac{2}{{V_{1} }} + \frac{1}{{V_{\text{B}} }} + \frac{{\beta_{1} }}{{V_{\text{A}} V_{1} V_{\text{B}} }})]t}} ,\,\,\gamma_{4} = {\text{e}}^{{ - [\lambda + \alpha (\frac{1}{{V_{\text{A}} }} + \frac{2}{{V_{1} }} + \frac{1}{{V_{\text{B}} }} - \frac{{\beta_{1} }}{{V_{\text{A}} V_{1} V_{\text{B}} }})]t}} ,\,{\text{and}}\,\,\alpha = \frac{{A_{\text{e}} D}}{L}. \hfill \\ \end{gathered} $$

#### VC–VC method for estimating D

A simple formula for estimating the diffusion coefficient with the decay effect was presented in the Ref. [9] as follows:

34 $$ D = \frac{s - \lambda }{{\frac{{A_{\text{e}} }}{L}\left( {\frac{1}{{V_{\text{A}} }} + \frac{1}{{V_{\text{B}} }}} \right)}} $$

where *s* is a constant slope of the plot of $\ln \left[ {\frac{{C_{\text{o}} }}{{C_{\text{A}} (t) - C_{\text{B}} (t)}}} \right]$ against t.

In this study, we also proposed two simple formulas for estimating D from the concentration distribution in the IR and the OR.

Assuming that a thinner specimen is used, the thickness term of the specimen can be ignored, and the asymptotic concentration distribution in the IR and OR can be gained using a Laplace inverse transform, as follows:

In the IR:

35 $$ C_{\text{A}} = \frac{{V_{\text{A}} }}{{V_{\text{A}} + V_{\text{B}} }}C_{\text{o}} {\text{e}}^{ - \lambda t} \left( {1 + \frac{{V_{\text{B}} }}{{V_{\text{A}} }}{\rm e}^{ - \gamma t} } \right) $$

In the OR:

36 $$ C_{\text{B}} = \frac{{V_{\text{A}} }}{{V_{\text{A}} + V_{\text{B}} }}C_{o} {\text{e}}^{ - \lambda t} \left( {1 - {\text{e}}^{ - \gamma t} } \right) $$

where $$ \gamma = \frac{{A_{\text{e}} D}}{L}(\frac{1}{{V_{\text{A}} }} + \frac{1}{{V_{\text{B}} }}) $$.

Rearranging these equations provides the simplified formulas as the following expressions:

In the IR:

37 $$ D = - \frac{{s_{\text{A}} \cdot L}}{{A_{\text{e}} }}\frac{{V_{\text{A}} V_{\text{B}} }}{{(V_{\text{A}} + V_{\text{B}} )}} $$

where *s*_A_ is a constant slope of the plot of $\ln \left[ {\left( {\frac{{V_{\text{A}} }}{{V_{\text{B}} }} + 1} \right)\frac{{C_{\text{A}} }}{{C_{\text{o}} {\text{e}}^{ - \lambda t} }} - 1} \right]$ against t.

In the OR:

38 $$ D = - \frac{{s_{\text{B}} \cdot L}}{{A_{\text{e}} }}\frac{{V_{\text{A}} V_{\text{B}} }}{{(V_{\text{A}} + V_{\text{B}} )}} $$

where *s*_B_ is a constant slope of the plot of $\ln \left[ {1 - \left( {\frac{{V_{\text{B}} }}{{V_{\text{A}} }} + 1} \right)\frac{{C_{\text{B}} }}{{C_{\text{o}} {\text{e}}^{ - \lambda t} }}} \right]$ against t.

## Model verification

### Comparison with VC–VC model

**Fig. 2:**
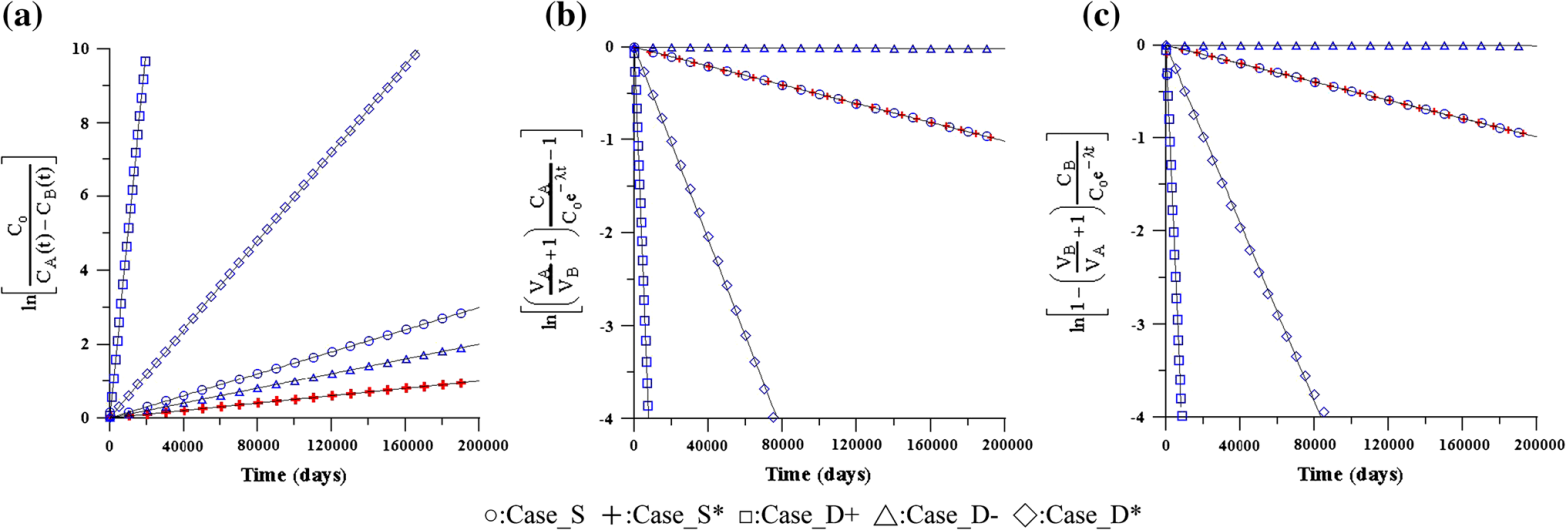
Estimation of D for Case_S, Case_S*, Case_D+, Case_D−, and Case_D* by Eqs. (34, 37, 38). **a** VC–VC method by Eq. (34), **b** VC–VC IR method by Eq. (37), **c** VC–VC OR method by Eq. (38). *White circle*:Case_S, *plus sign*:Case_S*, *white square*:Case_D+, *white triangle*:Case_D−, *white* *diamond*:Case_D*

In this section, we first validated the proposed simple formulas Eq. (37), called VC–VC IR method, and Eq. (38), called VC–VC OR method, to estimate D in the VC–VC model by calculating the default values of five cases (Case_S*, Case_S, Case_D+, Case_D*, and Case_D−), as shown in Table 1 and Fig. 2, and compared them with the results of Eq. (34). After plotting the linear relationship against time (t), we acquired an approximate slope by using a linear regression. The experimental diffusion coefficients were obtained by inputting the approximate slope into Eqs. (34), 37, and 38). By comparing the obtained diffusion coefficients with the theoretical coefficients (Table 2), we could assess the validity of the proposed models.

**Table 1:** Default values employed in this study

	*L* (cm)	*A*_e_ (cm^2^)	*V*_A_ (cm^3^)	*V*_B_ (cm^3^)	*D* (cm^2^/day)	*λ* (1/day)
Case_S*	0.2	5	100	100	1E−5	1E−20
Case_S						1E−5
Case_D+					1E−3	
Case_D*					1E−4	
Case_D−					1E−7

**Table 2:** Estimation of D for five assumed cases

	Case_D+			Case_D*			Case_S			Case_S*			Case_D−		
Designed D	**1.000E** **−** **3**			**1.000E**−**4**			**1.000E**−**5**			1.000E−5			**1.000E**−**7**		
Value of *λ*	1.000E−5			1.000E−5			**1.000E**−**5**			**1.000E**−**20**			1.000E−5		
Estimated method	Eq. (34)	Eq. (37)	Eq. (38)	Eq. (34)	Eq. (37)	Eq. (38)	Eq. (34)	Eq. (37)	Eq. (38)	Eq. (34)	Eq. (37)	Eq. (38)	Eq. (34)	Eq. (37)	Eq. (38)
Fitted slope	5.078E−4	−5.112E−4	−4.887E−4	5.995E−5	−5.174E−5	−4.785E−5	1.502E−5	−5.089E−6	−4.930E−6	5.017E−6	−5.079E−6	−4.930E−6	1.007E−5	−8.422E−8	−2.533E−8
Estimated D	0.996E−3	1.022E−3	0.977E−3	0.999E−4	1.035E−4	0.957E−4	1.003E−5	1.016E−5	0.986E−5	1.003E−5	1.016E−5	0.986E−5	1.499E−7	1.684E−7	0.507E−8
Deviation (%)	−0.44	+2.24	−2.27	−0.11	+3.49	−4.31	+0.33	+1.59	−1.40	+0.33	+1.59	−1.40	+49.87	+68.44	−49.35

In these cases, the value of the error from the estimated D using the proposed formulas Eqs. (37) and (38) are slightly higher than the results calculated using Eq. (34). However, they are in a reasonable error range, except Case_D−. Because of the comparatively smaller diffusion coefficient in Case_D−, significantly fewer masses diffused into the OR; the lower concentration in the OR led to higher calculation errors.

From Case_S*, which is deliberately set as an extremely small decay constant (1E−20 day^−1^) to ignore the decay effect, we demonstrated that the proposed formula can be used to calculate the diffusion coefficient without the decay effect. In this case, we also proved that in a diffusion experiment, Eq. (37) can be used as the concentration distribution in IR, and Eq. (38) can be used as the concentration distribution in OR to help calculate D.

### Comparison with actual diffusion experiment

In this section, two actual through-diffusion experiments were adopted to verify our models. First experiment is the diffusion of ^125^I radioactive nuclide through granite core sample, which was described in the Ref. [15]. The other experiment was done in the Ref. [16]. In this experiment, the diffusion of radioactive U, Pu and Am carbonate complexes through Inada granite was performed.

1. Diffusion coefficient determination from data reported by Ref. [15]

The ^125^I nuclide with a half-life of 60.14 days is a tracer in the through-diffusion experiment conducted by Ref. [15] for determining its effective diffusion coefficient in Beishan granite. The parameter values of this experiment are listed in Table 3. Three experiments with varying sample thickness and outlet cell volume were performed. Four different analytical methods were applied to calculate effective diffusion coefficient value. The results are also shown in Table 3. According to the experimental design, the volume of injective reservoir (~1,800 mL) is larger than the volume of DR (~60 mL), suggesting that it is a CC–VC model. We redrew the experimental data of Fig. 5 reported in the Ref. 15 and analyzed it by CC–VC method. After plotting $\ln \left[ {1 - \frac{{C_{\text{B}} }}{{C_{\text{o}} }}{\text{e}}^{\lambda t} } \right]$ against time, a constant slope value can be obtained for calculating D by CC–VC method (Eq. 26). Since the experimental data reported by Lu et al. [15] provide insufficient information on which cell number is used, it is assumed that the slope of fitting result suits all three experiments. The estimated results are shown in Table 3. Our results of Cell 1 and Cell 2 are consistent with those reported in the Ref. [15].

**Table 3:** Experimental values reported by Lu et al. [15]

Cell number	*V*_A_ (cm^3^)	*V*_B_ (cm^3^)	Thickness (L, mm)	Effective cross section (A_e_, cm^2^)	Effective diffusion coefficient (*D*_e_, m^2^/s)				
					Analytical solution	Asymptotic plot	Numerical solution	Wolfrum’s plot	CC–VC method
1	1,800	62.40 ± 0.05	5.00 ± 0.05	15.4 ± 0.4	(2.70 ± 0.01)E−12	(2.45 ± 0.02)E−12	(2.74 ± 0.02)E−12	(2.61 ± 0.01)E−12	*2.47E* *−* *12*
2		61.83 ± 0.05	4.85 ± 0.05		(2.40 ± 0.01)E−12	(2.22 ± 0.02)E−12	(2.57 ± 0.02)E−12	(2.26 ± 0.01)E−12	*2.37E* *−* *12*
3		57.15 ± 0.05	9.95 ± 0.05		(2.78 ± 0.01)E−12	(2.66 ± 0.02)E−12	(2.84 ± 0.02)E−12	(2.73 ± 0.01)E−12	*4.50E* *−* *12*

(2) Diffusion coefficient determination from data reported by Ref. [16]

The ^233^U, ^239^Pu and ^241^Am radioactive nuclides were prepared as an injective source for through-diffusion experiment to determine effective diffusion coefficient in biotitic granite. The experiment was performed in triplicate using three granite disks, C15, C17 and C19. The parameter values of the experiment are summarized in Table 4. The volumes of IR and OR in this through-diffusion experiment are the same, which makes the experimental concept similar to a VC–VC model. Since the concentration of source was kept constant during the experiment, the CC–VC method is suitable for analyzing the concentration distribution in the OR. However, the VC–VC method was also applied to determine the diffusion coefficient for comparison.

**Table 4:** Experimental values reported by Yamaguchi and Nakayama [16]

Nuclide	Half life (*H*_f_, year)	Cell number	*V*_A_ (cm^3^)	*V*_B_ (cm^3^)	Thickness (L, mm)	Diameter (mm)	Porosity (*n*)^a^	Rock capacity factor	Effective diffusion coefficient (*D*_e_, m^2^/s)			
									Crank method	CC–VC method	VC–VC method	VC–VC OR method
Uranium (^233^U)	1.59E+5	C15	116	116	5	40	0.49 ± 0.07 %	0.266 ± 0.047	(1.56 ± 0.06)E−13	*3.28E* *−* *14*	*8.89E* *−* *13*	*1.79E* *−* *12*
		C17						0.323 ± 0.034	(1.43 ± 0.05)E−13	*4.75E* *−* *14*	*1.56E* *−* *12*	*3.14E* *−* *12*
		C19						0.37 ± 0.08	(1.28 ± 0.15)E−13	*3.37E* *−* *14*	*1.27E* *−* *12*	*2.55E* *−* *12*
Plutonium (^239^Pu)	2.41E+4	C15						0.125 ± 0.006	(6.94 ± 0.11)E−14	*5.31E* *−* *15*	*6.58E* *−* *14*	*1.34E* *−* *13*
		C17						0.125 ± 0.007	(5.34 ± 0.12)E−14	*7.86E* *−* *15*	*9.78E* *−* *14*	*1.98E* *−* *13*
		C19						0.085 ± 0.007	(2.95 ± 0.18)E−14	*1.90E* *−* *15*	*1.58E* *−* *14*	*3.25E* *−* *14*

^a^Reference [17]

**Fig. 3:**
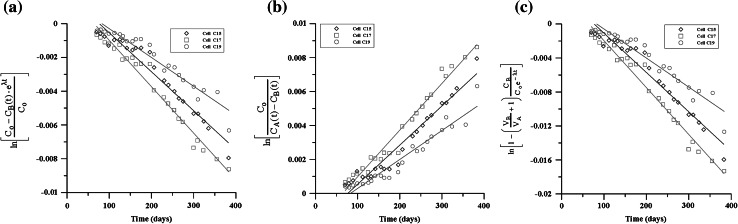
Estimation of D by three distinct analysis methods with identical uranium concentration. **a** By CC–VC method (Eq. (26)), **b** by VC–VC method (Eq.(34)), **c** by VC–VC OR method (Eq. (38))

**Fig. 4:**
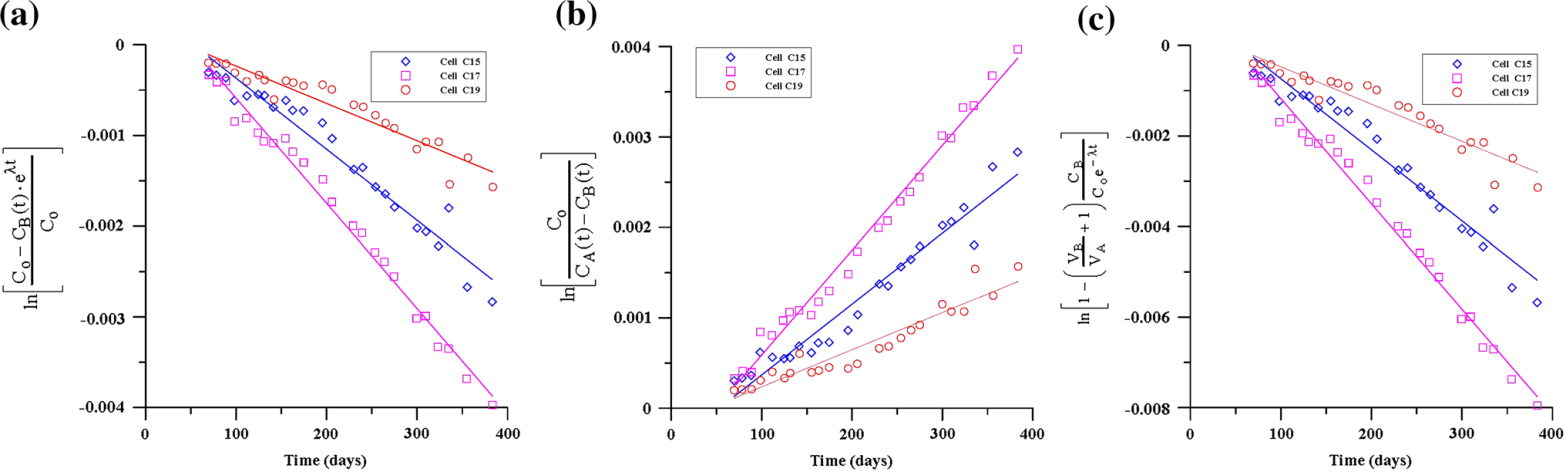
Estimation of D by three distinct analysis methods with identical plutonium concentration. **a** By CC–VC method (Eq. (26)), **b** by VC–VC method (Eq. (34)), **c** by VC–VC OR method (Eq. (38))

As described by Ref. [16], no diffusion of americium through the granite was detected. The experimental data of uranium and plutonium were obtained by redrawing Fig. 2. The diffusion coefficients of uranium and plutonium were estimated by three districted methods (i.e. the CC–VC method, the VC–VC method and the VC–VC OR method). The analysis results of uranium are shown in Fig. 3. Figure 4 shows the analysis results of plutonium. In Figs. 3 and 4, the concentrations distributed linearly with time for all three cells obviously. The apparent diffusion coefficients were estimated by each analytical method according to each constant slope. Then, the apparent diffusion coefficient was converted to effective diffusion coefficient by considering porosity and rock capacity factor. The calculated effective diffusion coefficients are shown in Table 4. Generally, the values analyzed in this study by CC–VC method are slightly lower, while the results estimated by VC–VC method and VC–VC OR method are somewhat larger comparing with the values reported by Ref. [16] using Crank method. Nevertheless, the obtained values by our proposed method are within experimental error.

## Analysis and discussion

### Model differences

Laboratory technicians are experts in the experimental method and strive to reduce the number of experimental errors. However, they may make some analytical mistakes because they may adopt an inappropriate method to interpret their data. In this section, we employ Case_S and Case_D+ to validate the CC–CC method (Eq. (13)) and CC–VC method (Eq. (26)) we proposed, and also to discuss what might happen when D is estimated using an unsuitable method.

**Fig. 5:**
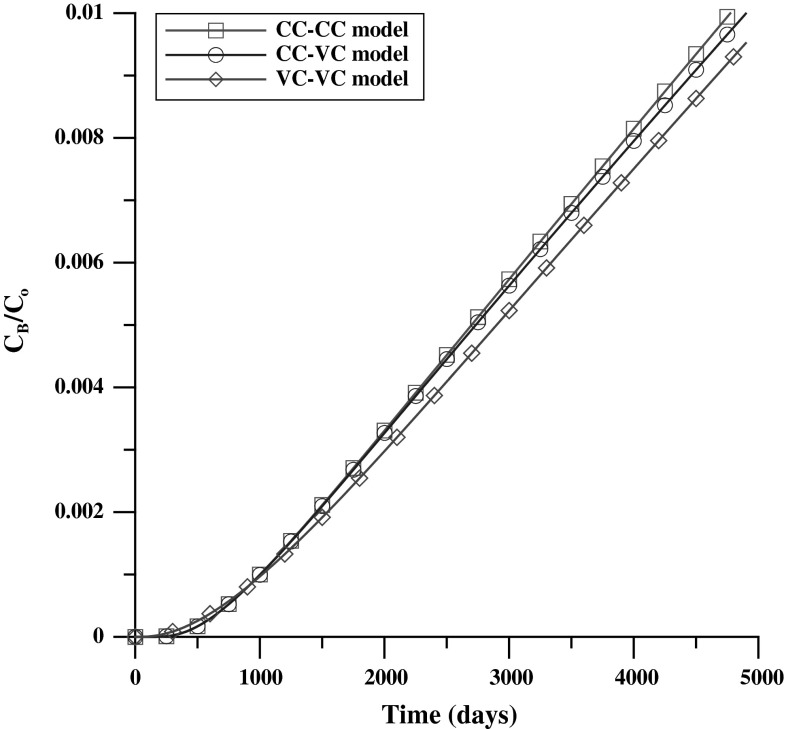
The Case_S concentration distribution in the OR of CC–CC, CC–VC and VC–VC models

**Fig. 6:**
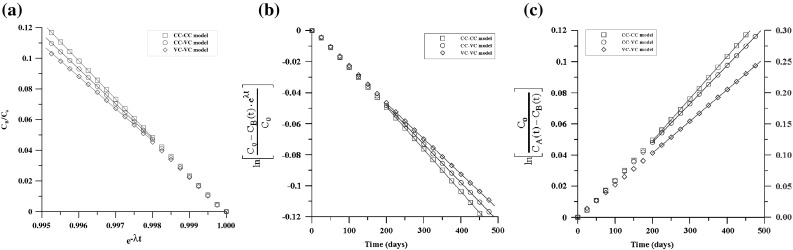
Estimation of D by three distinct analysis methods of Case_S. **a** by CC–CC method (Eq. (13)), **b** by CC–VC method (Eq. (26)), **c** by VC–VC method (Eq. (34))

First, concentration distributions of Case_S were obtained from three numerical experiments of the CC–CC model (Eq. (8)), CC–VC model (Eq. (18)), and the VC–VC model (Eq. (31)). The diffusion coefficient was then estimated using three distinct analysis methods (i.e., the CC–CC method, the CC–VC method, and the VC–VC method was estimated using Eq. (34)). Figure 5 shows that the concentration distribution in the OR was comparable between numerical experiments. An approximate slope can be derived from the analysis methods and the estimated D can be obtained, except in the case of the VC–VC numerical experiment, which involved estimating D by using the CC–CC method or CC–VC method, as shown in Fig. 6 and Table 5. In that case, an unreasonable result may have been obtained.

**Table 5:** Estimation of D for Case_S from three distinct numerical experiments by three analysis methods

Designed D	1.000E−5								
Numerical experiment	CC–CC model			CC–VC model			VC–VC model		
Analysis method	CC–CC method	CC–VC method	VC–VC method	CC–CC method	CC–VC method	VC–VC method	CC–CC method	CC–VC method	VC–VC method
Fitted slope	*−2.497E*−*1*	−2.402E*−*1	2.345E−1	−2.586E−6	*−2.489E*−*6*	−2.425E−6	2.429E−6	2.337E−6	*1.502E* *−* *5*
Estimated D	*9.988E* *−* *6*	9.609E−6	9.382E−6	1.034E−5	*9.955E*−*6*	9.701E−6	−**3.028E**−**5**	−**3.065E**−**5**	*1.003E* *−* *5*
Deviation (%)	*−0.12*	−3.91	−6.18	+3.44	−*0.45*	−2.99			*+0.33*

**Fig. 7:**
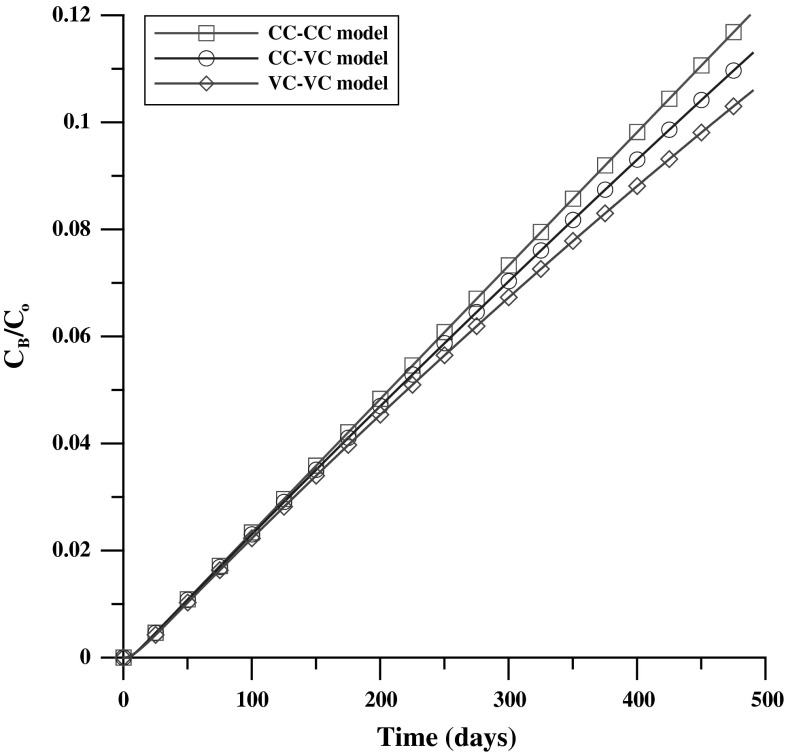
The Case_D+ concentration distribution in the OR of CC–CC, CC–VC and VC–VC models

**Fig. 8:**
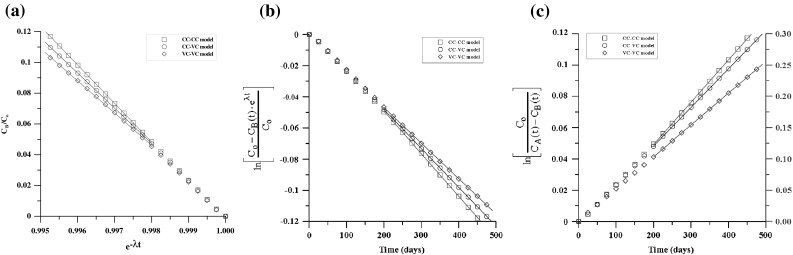
Estimation of D by three distinct analysis methods of Case_D+. **a** By CC–CC method (Eq. (13)), **b** by CC–VC method (Eq. (26)), **c** by VC–VC method (Eq. (34))

With a higher diffusion coefficient (Case_D+), the concentration distributions in the OR seem to depart from each numerical experiment (Fig. 7). After arranging for linear regression against time, the differences are obvious between each experiment (Fig. 8). A higher deviation obtained using an unsuitable analysis method is displayed clearly, as shown in Table 6.

**Table 6:** Estimation of D for Case_D+ from three distinct numerical experiments by three analysis methods

Designed D	1.000E−3								
Numerical experiment	CC–CC			CC–VC			VC–VC		
Analysis method	CC–CC method	CC–VC method	VC–VC method	CC–CC method	CC–VC method	VC–VC method	CC–CC method	CC–VC method	VC–VC method
Fitted slope	−*2.500E*+*1*	−2.283E+1	−2.094E+1	−2.729E−4	−*2.492E*−*4*	−2.276E−4	2.722E−4	2.474E−4	*5.078E−4*
Estimated D	*1.000E*−*3*	9.130E−4	8.377E−4	1.092E−3	*9.968E*−*4*	9.105E−4	1.049E−3	9.495E−4	*0.996E−3*
Deviation (%)	*0.00*	−8.70	−16.23	+9.16	−*0.32*	−8.95	+4.88	−5.05	−*0.44*

Tables 5 and  6 show that using CC–CC method (Eq. (13)) and CC–VC method (Eq. (26)) to determine diffusion coefficient of CC–CC model and CC–VC model can have a reasonable value of diffusion coefficient. The derivation is <0.45 %. That proves our proposed method can calculate the diffusion coefficient with the decay effect. The analysis results also show that if an unsuitable analysis method is applied for the diffusion experiment, a similar value may be estimated. However, the parameter should be estimated using the correct analysis method for obtaining the lowest deviation.

### Volume ratio of IR/OR

The CC–VC model is a special case of the VC–VC model. In the VC–VC model, if the concentration reduction in the IR can be ignored, the VC–VC model can be considered a CC–VC model. This condition is often based on the IR relative to the OR, which contains large amounts of nuclides. In the experiment, the IR is designed with a larger volume, solutes are supplied circularly, or only a few nuclides diffused from the IR to the OR within the experiment period. This section clarifies which condition of the VC–VC model can be simplified to obtain the CC–VC model. In addition, the accuracy of the estimated D should be an evaluation criterion.

**Fig. 9:**
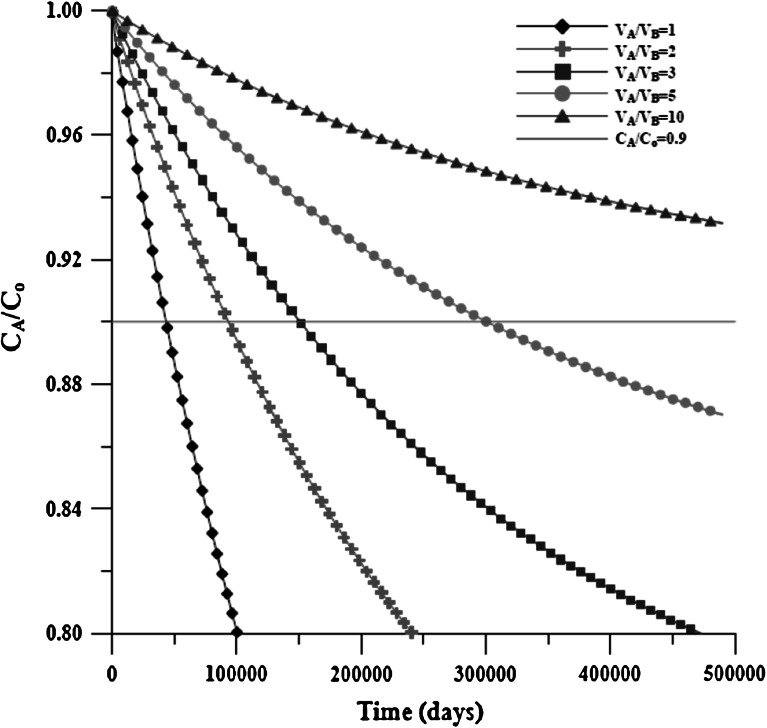
Concentration distribution of Case_S* with varied volume ratio in the IR

First, we used Case_S* to ignore the decay effect. Because the nuclide concentration is reduced with the decay effect in the IR or in the OR, confirming whether the concentration in the IR stays constant is difficult. The concentration distributions in the IR with a distinct volume ratio (*V*_B_/*V*_A_ = 1/1, 1/2, 1/3, 1/5, 1/10) are shown in Fig. 9. Only the declined concentration of the volume ratio of 1/10 in the IR is more than 90 % of the source concentration during the numerical experiment time. The concentration in the IR is reduced more slowly with a higher volume ratio. The reduced concentration in the IR of higher than 90 % is assumed to be a constant concentration source; otherwise, it is a variable concentration source. Figure 9 shows that the concentration in the IR is reduced to 90 % at 4.46E+4 days of the volume ratio of 1. The time required for the concentration to be reduced to 90 % can be estimated with the following formula, which is derived from Eq. (35).

39 $$ T_{C} = \frac{1}{{\alpha_{{V_{\text{B}} }} \left( {1 + R_{{V_{\text{OR}} /V_{\text{IR}} }} } \right)}}\ln \left[ {\frac{{R_{{V_{\text{OR}} /V_{\text{IR}} }} }}{{R_{{C_{\text{IR}} /C_{\text{o}} }} \left( {1 + R_{{V_{\text{OR}} /V_{\text{IR}} }} } \right) - 1}}} \right] $$

where $\alpha_{{V_{\text{B}} }}$ can be expressed as $\alpha_{{V_{\text{B}} }} = \frac{\alpha }{{V_{\text{B}} }} = \frac{{A_{\text{e}} D}}{{V_{\text{B}} L}}$, $R_{{V_{\text{OR}} /V_{\text{IR}} }}$ is the volume ratio of OR and IR, and $R_{{C_{\text{IR}} /C_{\text{o}} }}$ is the ratio of the declined concentration and initial concentration in the IR. In this study, $R_{{C_{IR} /C_{o} }}$ is assumed to be 0.9.

The T_C_s estimated using (39) are 9.51E+4 days, 1.53E+5 days, and 2.22E+5 days, when $R_{{V_{\text{OR}} /V_{\text{IR}} }}$ is 1/2, 1/3, and 1/5, respectively. We calculated D by using the VC–VC method and the CC–VC method with the two conditions (i.e., *t* < *T*_C_ or *t* > *T*_C_). The results are shown in Table 7. The estimated D using the CC–VC model of *t* < *T*_C_ clearly has a deviation lower than that of *t* > *T*_C_, except when $R_{{V_{\text{OR}} /V_{\text{IR}} }}$ is 1/10, in which case *C*_A_/*C*_o_ is larger than 0.9 for the duration of the numerical experiment. The deviation of the estimated D using the CC–VC model of *t* < *T*_C_ is approximately <7 %, which shows that a reasonable result can be achieved when the diffusion experiment is established as a VC–VC model but analyzed using the CC–VC method when the time t is <*T*_C_. However, the estimated D using the VC–VC method has a smaller deviation than using the CC–VC method. This result shows that an experiment with a suitable analysis method should yield a more accurate parameter.

**Table 7:** Estimation of D by CC–VC and VC–VC methods

Designed D	1.000E−5														
Volume ratio ($R_{{V_{OR} /V_{IR} }}$)	1/1			1/2			1/3			1/5			1/10		
Analyticals method	CC–VC method		VC–VC method	CC–VC method		VC–VC method	CC–VC method		VC–VC method	CC–VC method		VC–VC method	CC–VC method		VC–VC method
	*t* < *T*_C_	*t* > *T*_C_		*t* < *T*_C_	*t* > *T*_C_		*t* < *T*_C_	*t* > *T*_C_		*t* < *T*_C_	*t* > *T*_C_		*t* < *T*_C_	*t* > *T*_C_	
Estimated D	9.399E−6	8.722E−6	9.992E−6	9.349E−6	8.366E−6	9.990E−6	9.297E−6	8.019E−6	9.986E−6	9.338E−6	8.860E−6	9.982E−6	9.434E−6	9.434E−6	9.977E−6
Deviation (%)	−6.01	−12.78	−0.08	−6.51	−16.34	−0.10	−7.03	−19.81	−0.14	−6.62	−11.40	−0.18	−5.66	−5.66	−0.23

**Fig. 10:**
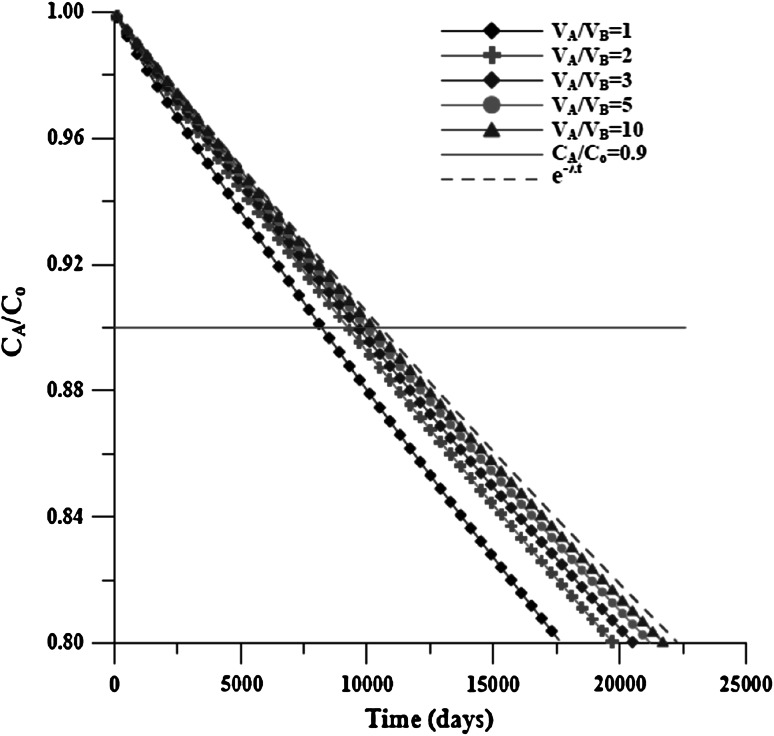
Concentration distribution of Case_S with varied volume ratio in the IR

Using Case_S for the case with the decay effect, the concentration distributions in the IR with distinct $R_{{V_{\text{OR}} /V_{\text{IR}} }}$ are shown in Fig. 10. For example, when $R_{{V_{\text{OR}} /V_{\text{IR}} }}$ is 1/1, the time is <1.00E+4, because *C*_A_/*C*_o_ = 0.9. It is difficult to distinguish whether the diffusion effect or the decay effect cause a reduction in concentration. The concentration data product ${\text{e}}^{\lambda t}$ used to eliminate the decay effect can be used to obtain the concentration data without the decay effect, as shown in Fig. 9. The *T*_C_ can then be determined with Eq. (39), and the same result can be obtained, as noted previously.

## Conclusion

In this study, first two simplified formulas of the VC–VC model, which were used to gain another two diffusion coefficient data from VC–VC diffusion experiment, were proposed and verified. That benefits to cross comparison of the estimated results and enhances experiment effectiveness.

Then, our proposed methods for estimating D were verified by using actual through-diffusion experimental data. These experiments were carried out with short half life nuclide such as ^125^I (60.14 days) and long half life nuclide such as ^233^U (1.59E+5 years) and ^239^Pu (2.41E+4 years). The results indicated that our proposed methods with decay effect are practical.

A CC–CC model and a VC–CC model with decay effect were developed. The proposed simplified formulas for determining diffusion coefficient were validated. These two models and analysis methods made up the lack of 1-D through diffusion model with decay effect. Numerically distinct through-diffusion experiments were analyzed using varied methods to investigate the deviation of the diffusion coefficient with the unsuitable analysis methods proving that performing a diffusion experiment with a correct analysis method is necessary. Three through-diffusion models and the proposed methods for estimating apparent diffusion coefficient are summarized in Table 8.

**Table 8:** Summary of three through-diffusion models and proposed methods for D

Diffusion model	Equation	Plotting	D
CC–CC model	$\frac{C}{{C_{o} }} = \left( {1 - \frac{x}{L} - \sum\limits_{n = 1}^{\infty } {\frac{2}{n\pi }\sin \frac{n\pi x}{L}{\text{e}}^{{ - \frac{{Dn^{2} \pi^{2} }}{{L^{2} }}t}} } } \right){\text{e}}^{ - \lambda t}$	CC–CC method	$D = - \frac{{s\lambda V_{B} L}}{{A_{e} }}$
	$\frac{{C_{B} }}{{C_{o} }} = \frac{{DA_{e} }}{{V_{B} L}}\left\{ { - \frac{1}{\lambda }{\text{e}}^{ - \lambda t} + \frac{L}{{2\sqrt {\lambda D} }}\left[ {\coth \left( {\frac{L}{2}\sqrt {\frac{\lambda }{D}} } \right) - \tanh \left( {\frac{L}{2}\sqrt {\frac{\lambda }{D}} } \right)} \right] - 2\sum\limits_{n = 1}^{\infty } {\frac{{( - 1)^{n} }}{{\frac{{Dn^{2} \pi^{2} }}{{L^{2} }} + \lambda }}{\text{e}}^{{ - \left( {\frac{{Dn^{2} \pi^{2} }}{{L^{2} }} + \lambda } \right)t}} } } \right\}$	$\frac{{C_{B} }}{{C_{o} }}$ v.s. $e^{ - \lambda t}$	
CC–VC model	(1) Semi-analytical solution	CC–VC method	$D = - \frac{{sV_{B} L}}{{A_{e} }}$
	$\frac{{\overline{C} }}{{C_{o} }} = \frac{1}{p + \lambda }\left[ {\cosh (mx) - \frac{M\sinh (mL) + \cosh (mL)}{M\cosh (mL) + \sinh (mL)}\sinh (mx)} \right]$, $\frac{{\overline{C}_{B} }}{{C_{o} }} = \frac{1}{p + \lambda }\frac{M}{M\cosh (mL) + \sinh (mL)}$ where $M = \frac{{mA_{e} D}}{{V_{B} (p + \lambda )}}$ and $m = \sqrt {\frac{p + \lambda }{D}}$	$\ln \left[ {1 - \frac{{C_{B} }}{{C_{o} }}{\text{e}}^{\lambda t} } \right]$ v.s. t	
	(2) Analytical solution		
	$\frac{C}{{C_{o} }} = {\text{e}}^{ - \lambda t} + \frac{{\alpha V_{1} }}{2\beta }\left( {\gamma_{1} - \gamma_{2} } \right) - \frac{1}{2}\left( {\gamma_{1} + \gamma_{2} } \right)$, $\frac{{C_{B} }}{{C_{o} }} = {\text{e}}^{ - \lambda t} + \frac{\alpha }{2\beta }\left( {V_{1} + 2V_{B} } \right)\left( {\gamma_{1} - \gamma_{2} } \right) - \frac{1}{2}\left( {\gamma_{1} + \gamma_{2} } \right)$ where $\gamma_{1} = {\text{e}}^{{ - \left( {\lambda + \frac{2\alpha }{{V_{1} }} + \frac{\alpha }{{V_{B} }} + \frac{\beta }{{V_{1} V_{B} }}} \right)t}}$, $\gamma_{2} = {\text{e}}^{{ - \left( {\lambda + \frac{2\alpha }{{V_{1} }} + \frac{\alpha }{{V_{B} }} - \frac{\beta }{{V_{1} V_{B} }}} \right)t}}$, and $\beta = \sqrt {\alpha^{2} \left( {V_{1}^{2} + 4V_{B}^{2} } \right)}$		
VC–V	(1) Semi-analytical solution	(1) VC–VC method	$D = \frac{s - \lambda }{{\frac{{A_{e} }}{L}\left( {\frac{1}{{V_{A} }} + \frac{1}{{V_{B} }}} \right)}}$
C model	$\frac{{\overline{C}_{A} }}{{C_{o} }} = \frac{{M_{2} V_{A} }}{{(p + \lambda )M_{2} V_{A} - (p + \lambda )M_{1} V_{B} }}$, $\frac{{\overline{C}_{B} }}{{C_{o} }} = \frac{{V_{A} }}{{(p + \lambda )M_{2} V_{A} - (p + \lambda )M_{1} V_{B} }}\left[ {M_{2} \cosh (m_{1} L) + \sinh (m_{1} L)} \right]$ where $M_{2} = - \frac{{M_{1} \cosh (m_{1} L) + \sinh (m_{1} L)}}{{M_{1} \sinh (m_{1} L) + \cosh (m_{1} L)}}$, $M_{1} = \frac{{m_{1} A_{e} D}}{{V_{B} (p + \lambda )}}$, and $m_{1} = \sqrt {\frac{p + \lambda }{D}}$.	$\ln \left[ {\frac{{C_{o} }}{{C_{A} (t) - C_{B} (t)}}} \right]$ v.s. t	
		(2) VC–VC IR method	$D = - \frac{{s_{A} \cdot L}}{{A_{e} }}\frac{{V_{A} V_{B} }}{{(V_{A} + V_{B} )}}$
		$\ln \left[ {\left( {\frac{{V_{A} }}{{V_{B} }} + 1} \right)\frac{{C_{A} }}{{C_{o} e^{ - \lambda t} }} - 1} \right]$ v.s. t	
	(2) Analytical solution		
	$$ C_{A} = \frac{{V_{A} C_{o} }}{V}\left\{ {{\text{e}}^{ - \lambda t} + \frac{{\left( {\gamma_{3} - \gamma_{4} } \right)\left( { - V_{1}^{2} V_{A} + 2V_{1}^{2} V_{B} - V_{1} V_{A} V_{B} + V_{1} V_{B}^{2} - 2V_{A} V_{B}^{2} } \right)}}{{2\beta_{1} V_{A} }} + \frac{{\beta_{1} \left( {\gamma_{3} + \gamma_{4} } \right)\left( {V_{1} + V_{B} } \right)}}{{2\beta_{1} V_{A} }}} \right\} $$		
		(3) VC–VC OR method	$D = - \frac{{s_{B} \cdot L}}{{A_{e} }}\frac{{V_{A} V_{B} }}{{(V_{A} + V_{B} )}}$
	$C_{B} = \frac{{V_{A} C_{o} }}{V}\left\{ {{\text{e}}^{ - \lambda t} + \frac{{\left( {\gamma_{3} - \gamma_{4} } \right)\left( {V_{1} V_{A} + V_{1} V_{B} + 2V_{A} V_{B} } \right) - \beta_{1} \left( {\gamma_{3} + \gamma_{4} } \right)}}{{2\beta_{1} }}} \right\}$ where $V = V_{A} + V_{1} + V_{B}$, $\alpha = \frac{{A_{e} D}}{L}$, $\beta_{1} = \sqrt {V_{1}^{2} V_{A}^{2} - 2V_{1}^{2} V_{A} V_{B} + V_{1}^{2} V_{B}^{2} + 4V_{A}^{2} V_{B}^{2} }$,$\gamma_{3} = {\text{e}}^{{ - [\lambda + \alpha (\frac{1}{{V_{A} }} + \frac{2}{{V_{1} }} + \frac{1}{{V_{B} }} + \frac{{\beta_{1} }}{{V_{A} V_{1} V_{B} }})]t}}$, and $\gamma_{4} = {\text{e}}^{{ - [\lambda + \alpha (\frac{1}{{V_{A} }} + \frac{2}{{V_{1} }} + \frac{1}{{V_{B} }} - \frac{{\beta_{1} }}{{V_{A} V_{1} V_{B} }})]t}}$		
		$\ln \left[ {1 - \left( {\frac{{V_{B} }}{{V_{A} }} + 1} \right)\frac{{C_{B} }}{{C_{o} e^{ - \lambda t} }}} \right]$ v.s. t	

Finally, the research also discussed the concept of critical time (*T*_c_). If the operating time of the VC–VC diffusion experiment was <*T*_c_, it could be analyzed using the CC–VC method. Otherwise, it should analyze by VC–VC method. That is an important reference for laboratory technicians who want to calculate diffusion coefficient and decide the reasonable analysis method.
